# Association between D-dimer and long-term mortality in patients with acute severe hypertension visiting the emergency department

**DOI:** 10.1186/s40885-023-00244-7

**Published:** 2023-06-15

**Authors:** Byung Sik Kim, Jeong-Hun Shin

**Affiliations:** Division of Cardiology, Department of Internal Medicine, Hanyang University College of Medicine, Hanyang University Guri Hospital, 153 Gyeongchun-Ro, Guri, Gyeonggi-Do 11923 Republic of Korea

**Keywords:** d-dimer, Acute severe hypertension, Emergency department, Mortality

## Abstract

**Objective:**

High levels of d-dimer, a marker of thrombotic events, are associated with poor outcomes in patients with various cardiovascular diseases. However, there has been no research on its prognostic implications in acute severe hypertension. This study investigated the association between d-dimer levels and long-term mortality in patients with severe acute hypertension who visited the emergency department.

**Design and method:**

This observational study included patients with acute severe hypertension who visited the emergency department between 2016 and 2019. Acute severe hypertension was defined as a systolic blood pressure ≥ 180 mmHg or a diastolic blood pressure ≥ 100 mmHg. Among the 10,219 patients, 4,127 who underwent d-dimer assay were analyzed. The patients were categorized into tertiles based on their d-dimer levels at the time of emergency department admission.

**Results:**

Among the 4,127 patients with acute severe hypertension, 3.1% in the first (lowest) tertile, 17.0% in the second tertile, and 43.2% in the third (highest) tertile died within 3 years. After the adjustment for confounding variables, the third tertile of the d-dimer group (hazard ratio, 6.440; 95% confidence interval, 4.628–8.961) and the second tertile of the d-dimer group (hazard ratio, 2.847; 95% confidence interval, 2.037–3.978) had a significantly higher risk of all-cause mortality over 3 years than the first tertile of the d-dimer group.

**Conclusions:**

d-dimer may be a useful marker for identifying the risk of mortality among patients with acute severe hypertension who visit the emergency department.

**Supplementary Information:**

The online version contains supplementary material available at 10.1186/s40885-023-00244-7.

## Introduction

Acute severe hypertension is characterized by a sudden and marked increase in blood pressure (BP) in patients with or without a history of hypertension [1]. This acute elevation in BP can lead to progressive damage to the heart, brain, kidneys, and other blood vessels [2, 3], known as acute hypertension-mediated organ damage (HMOD) or thromboembolic complications, leading to increased morbidity and mortality rates [4, 5]. Although the prognosis of patients with acute severe hypertension has improved in recent years, mortality rates remain high [6–9]. Despite the high risk of mortality, studies are limited on prognostic factors of acute severe hypertension [10–15]. Therefore, identifying markers or developing tools for risk stratification is of utmost importance for these patients.d-dimer is a breakdown product of cross-linked fibrin generated during thrombus formation and may serve as a marker of thrombogenesis and hypercoagulability [16]. Increased d-dimer levels have been associated with adverse clinical outcomes [17] such as pulmonary embolism [18], venous thromboembolism [19], tumors [20], stroke [21], aortic dissection [22], and coronary artery disease [23]. Furthermore, d-dimer levels were higher in hypertensive individuals than in controls and increased significantly with hypertension severity [24]. However, limited data are available regarding the association between d-dimer levels and mortality in patients with acute severe hypertension. Therefore, this study aimed to evaluate the association between d-dimer levels and long-term mortality in patients with acute severe hypertension who visited the emergency department (ED).

## Methods

### Study participants

**Fig. 1:**
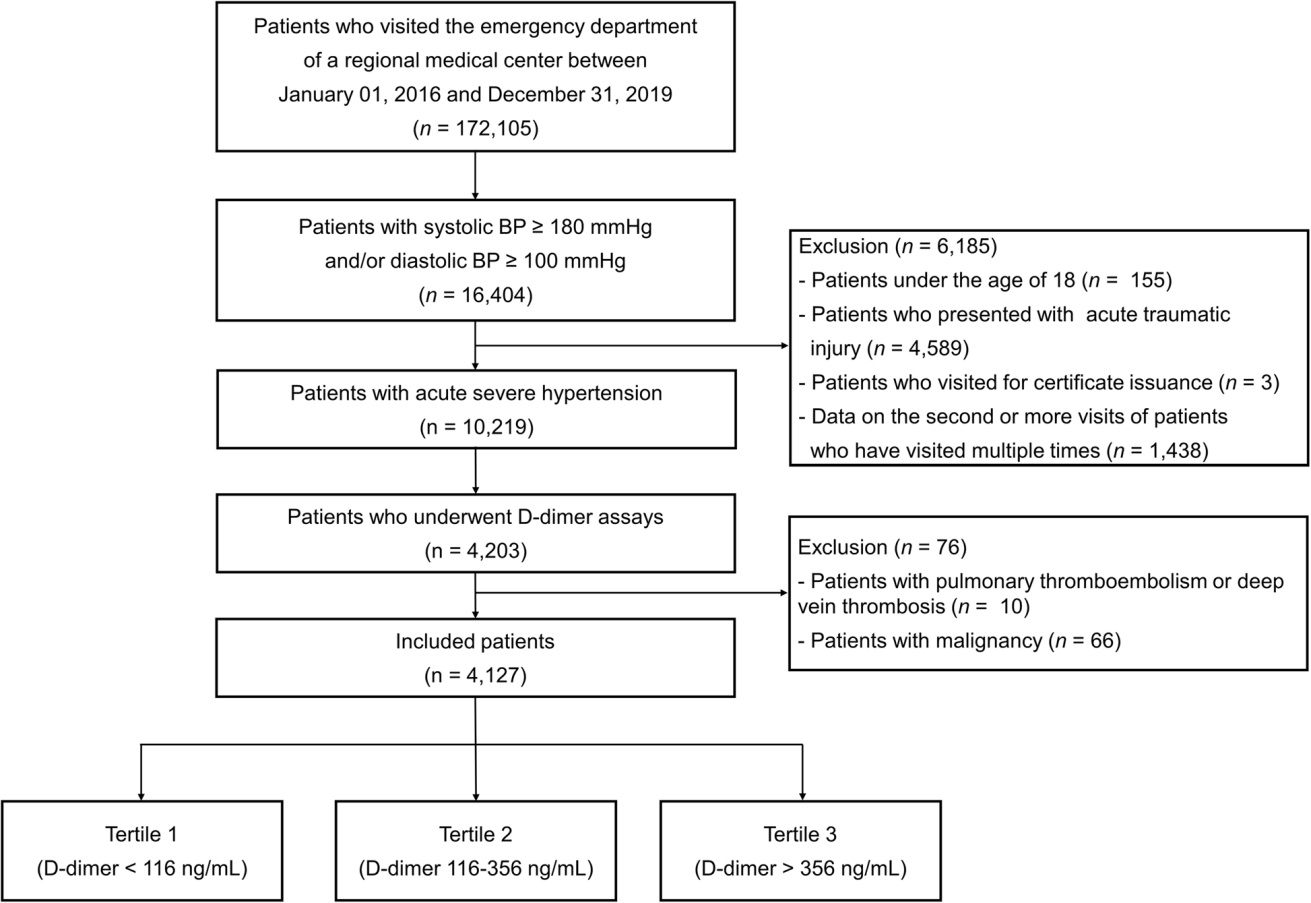
Study flowchart. BP, Blood pressure

This retrospective cohort study was conducted at a regional emergency medical center affiliated with Academic University Hospital in Guri-si, Gyeonggi-do, Korea. The study design, definitions of comorbidities, and primary results were published previously [7]. The medical records of 172,105 patients who visited the ED between January 2016 and December 2019 were reviewed. Of these, 16,404 patients were diagnosed with acute severe hypertension, defined as a systolic blood pressure (SBP) ≥ 180 mmHg or a diastolic blood pressure (DBP) ≥ 100 mmHg. Patients aged < 18 years, those with acute trauma, and those who visited for certification were excluded. Only the first visits of patients who visited the ED multiple times were included. Among 10,219 patients with acute severe hypertension, patients with pulmonary thromboembolism, deep vein thrombosis, or cancer, as well as those who had no d-dimer data during the ED visit were excluded. Finally, 4,127 patients were included in this study (Fig. 1). This study was conducted in compliance with the Declaration of Helsinki and was reviewed and approved by the Institutional Review Board of Hanyang University Guri Hospital, which waived the requirement for written informed consent due to the study’s retrospective design.

### d-dimer assay

d-dimer levels were measured using a Hemosil d-dimer HS on an ACL-TOP 550 automated coagulation analyzer according to the manufacturer’s instructions (Instrumentation Laboratory, Lexington, MA, USA). The patients were classified into three groups according to d-dimer level as follows: tertile 1, < 116 ng/mL; tertile 2, 116–356 ng/mL; and tertile 3, > 356 ng/mL.

### Data collection

The data were collected from the patients’ electronic medical records by an experienced data collector under the supervision of the principal investigator. The following demographic and clinical information was extracted: age, sex, initial BP, and traditional cardiovascular risk factors such as hypertension, diabetes mellitus, dyslipidemia, chronic kidney disease, end-stage renal disease, cigarette smoking, and alcohol consumption status. A history of ischemic or hemorrhagic stroke, heart failure, or coronary artery disease was also recorded. The following laboratory data were collected: d-dimer level, estimated glomerular filtration rate (eGFR), troponin-I level, and hemoglobin level. Additionally, diagnostic tests, such as a urine dipstick test, chest radiography, and electrocardiography (ECG), were performed to evaluate the presence of acute HMOD.

Data on events during the index hospitalization and follow-up periods (admission, discharge, ED visit, readmission, and death) were also collected. Mortality incidence and timing were extracted from the National Health Insurance Service in South Korea, while other event data were obtained from the electronic medical records.

### Definition

Acute HMOD was defined as the co-occurrence of acute heart failure, acute ischemic stroke, acute hypertensive encephalopathy, intracerebral hemorrhage, acute hypertensive retinopathy, acute coronary syndrome, acute renal failure, or acute aortic dissection [7]. Cardiomegaly was diagnosed when the ratio of maximum horizontal cardiac diameter to maximum horizontal inner thoracic cage diameter was > 0.5 on chest radiography [25]. The diagnosis of left ventricular hypertrophy (LVH) was made based on ECG findings that satisfied either the Sokolow-Lyon criterion (the sum of the amplitude of S in V1 and the amplitude of R in V5 or V6 must be ≥ 3.5 mV) or the Cornell voltage criterion (the sum of the amplitude of R in aVL and the amplitude of S or QS complex in V3 has a cutoff of > 2.8 mV in men and > 2.0 mV in women) [26]. BP was measured in the brachial artery using an automatic BP machine (Spot Vital Signs LXi; Welch Allyn, Skaneateles Falls, NY, USA) in the ED.

### Statistical analyses

Given the skewed distribution of all continuous variables as detected by the Kolmogorov–Smirnov test, continuous variables are presented as median and interquartile range. Categorical data are presented as frequency and percentage. The patients’ baseline characteristics were compared using the Kruskal–Wallis test, followed by Dunn’s multiple comparison test for continuous variables. In addition, the chi-squared or Fisher’s exact test was used to examine categorical variables. Kaplan–Meier survival analyses and log-rank tests were used to compare survival probability according to d-dimer level. The association between d-dimer level and 3-year all-cause mortality was determined using a Cox proportional hazards regression model with stepwise backward variable selection based on the Akaike information criterion. In addition to the univariate analysis, three adjusted models that considered other clinically relevant variables were used. In Model 1, age, sex, and BP were adjusted for as possible confounding variables. Model 2 included the factors used in Model 1 and a medical history of comorbidities (hypertension, diabetes mellitus, dyslipidemia, ischemic stroke, hemorrhagic stroke, coronary artery disease, heart failure, and chronic kidney disease). Model 3 included the factors used in Model 2 plus the components of subclinical HMOD (eGFR, hemoglobin level, cardiomegaly on chest radiography, LVH on ECG, myocardial ischemia on ECG, and atrial fibrillation on ECG).

Hazard ratios (HR) and 95% confidence intervals (Cis) were calculated for each Cox proportional hazard regression model. Variance Inflation Factors (VIFs) were calculated for all covariates retained in each adjusted model after the backward selection procedure; the models with all VIFs were low except for those within the d-dimer tertiles, indicating no problem with multicollinearity. The missing rates for the candidate variables are reported in Table S1. Variables with high missing rates, such as cigarette smoking (27.3%), alcohol consumption (26.8%), serum troponin (21.6%), and proteinuria (33.6%), were excluded from the adjusted variables in the Cox proportional hazards regression analysis. We addressed the issue of missing data by performing multiple imputations using chained equations. Table S1 compares the variables between the original and imputed datasets. We performed an additional subgroup analysis using multivariate Cox proportional hazards regression models for four binary specifications: age (≥ 65 or < 65 years), sex, eGFR, and the presence or absence of acute HMOD.

We also conducted a restricted cubic spline curve analysis to demonstrate the continuous adjusted association between d-dimer level and 3-year all-cause mortality. A time-dependent receiver operating characteristic curve analysis was used to summarize the discrimination potential of d-dimer levels for 3-year all-cause mortality. Statistical significance was set at *p* < 0.05. All statistical analyses were conducted using the open-source statistical software R (version 4.2.2, www.R-project.org) and R-studio (version 2022.12.0 + 353; www.rstudio.com) as well as statistical packages including rms, ggplot2, mice, survival, tableone, survminer, and timeROC.

## Results

### Baseline characteristics

This study analyzed data from 4,127 patients with a median follow-up period of 2.5 years (interquartile range, 1.5–3.8 years). The patients’ baseline characteristics were determined based on their d-dimer levels, which were then divided into tertiles (Table 1). The average patient age was 65 years, and 45.8% of them were women. patients with higher d-dimer levels were older (54 vs. 68 vs. 76 years, *p* < 0.001) and included a higher proportion of women (34.7% vs. 49.6% vs. 53.1%, *p* < 0.001). They also had a higher prevalence of acute HMOD (38.3% vs. 46.1% vs. 53.7%, *p* < 0.001), cardiovascular risk factors, and comorbidities such as hypertension, diabetes, stroke, heart disease, kidney disease, and end-stage renal disease. Additionally, patients with higher d-dimer levels had higher systolic BP and troponin-I levels and lower eGFR and hemoglobin levels. They were also more likely to have proteinuria, cardiomegaly on chest radiography, and LVH, myocardial ischemia, or atrial fibrillation on ECG.

**Table 1:** Patients’ baseline characteristics according to d-dimer tertile

	All patients (*N* = 4,127)	Tertile 1^*^ (*n* = 1,376)	Tertile 2^†^ (*n* = 1,376)	Tertile 3^‡^ (*n* = 1,375)	*p* value
Age	65 (53–78)	54 (45–62)^§¶^	68 (57–79)^↑^	76 (63–83)	< 0.001
Women	1,891 (45.8)	478 (34.7)	683 (49.6)	730 (53.1)	< 0.001
Medical history					
Hypertension	2,304 (57.1)	596 (44.3)	821 (61.0)	887 (66.1)	< 0.001
Diabetes mellitus	1,143 (28.7)	242 (18.3)	383 (28.7)	518 (39.0)	< 0.001
Dyslipidemia	453 (11.4)	171 (12.9)	164 (12.4)	118 (8.9)	0.002
Ischemic stroke	379 (9.5)	60 (4.5)	125 (9.4)	194 (14.7)	< 0.001
Hemorrhagic stroke	129 (3.3)	20 (1.5)	39 (2.9)	70 (5.3)	< 0.001
Coronary artery disease	464 (11.7)	103 (7.8)	176 (13.3)	185 (14.0)	< 0.001
Heart failure	234 (5.9)	18 (1.4)	82 (6.2)	134 (10.2)	< 0.001
Chronic kidney disease	368 (9.3)	19 (1.4)	107 (8.1)	242 (18.3)	< 0.001
End-stage renal disease	182 (4.6)	7 (0.5)	57 (4.3)	118 (8.9)	< 0.001
Social history					
Cigarette smoking	814 (27.1)	366 (39.8)	241 (24.0)	207 (19.3)	< 0.001
Alcohol consumption	1,078 (35.7)	489 (53.0)	341 (33.7)	248 (22.8)	< 0.001
Triage vitals					
SBP, mmHg	184 (165–200)	181 (163–196)^§¶^	186 (169–202)	185 (165–203)	< 0.001
DBP, mmHg	105 (100–113)	106 (101–114)^§¶^	104 (97–113)	103 (96–113)	< 0.001
Laboratory tests					
d-dimer, ng/mL	201 (85–504)	63 (38–85)^§¶^	201 (152–268)^↑^	773 (504–1817)	< 0.001
eGFR, mL/min/1.73 m^2^	85 (61–99)	98 (86–106)^§¶^	83 (63–97)^↑^	65 (34–86)	< 0.001
Hb, g/dL	13.7 (12.2–15.0)	14.7 (13.6, 15.7)^§¶^	13.7 (12.4–14.8)^↑^	12.5 (10.6–13.9)	< 0.001
BNP, pg/mL	75 (26–282)	23 (13–55)^§¶^	72 (32–232)^↑^	221 (80–770)	< 0.001
Troponin-I, ng/mL	0.01 (0.01–0.03)	0.01 (0.01–0.01)^§¶^	0.01 (0.01–0.03)^↑^	0.03 (0.01–0.06)	< 0.001
Urinalysis					
Proteinuria^a^	931 (34.0)	108 (13.5)	277 (30.6)	546 (52.8)	< 0.001
Chest radiography					
Cardiomegaly	576 (14.5)	120 (9.1)	207 (15.8)	249 (18.8)	< 0.001
Electrocardiography					
LVH	448 (11.4)	107 (8.2)	164 (12.4)	177 (13.5)	< 0.001
Myocardial ischemia	355 (9.0)	109 (8.4)	105 (8.0)	141 (10.8)	0.025
Atrial fibrillation	281 (7.1)	46 (3.5)	96 (7.3)	139 (10.6)	< 0.001
Acute HMOD, *n* (%)	1,900 (46.0)	527 (38.3)	635 (46.1)	738 (53.7)	< 0.001

Data are presented as *n* (%) or median (IQR) as appropriate

*BNP* B-type natriuretic peptide, *DBP* Diastolic blood pressure, *eGFR* Estimated glomerular filtration rate, *Hb* Hemoglobin, *HMOD* Hypertension-mediated organ damage, *IQR* Interquartile range, *LVH* Left ventricular hypertrophy, *SBP* Systolic blood pressure

^*^The range of d-dimer levels in tertile 1 was < 116 ng/mL

^†^The range of d-dimer levels in tertile 2 is 116–356 ng/mL

^‡^The range of d-dimer level in tertile 3 was > 356 ng/mL

^§^Post hoc *p*: tertile 1 versus tertile 2: statistically significant, *p* < 0.05

^¶^Post hoc *p*: tertile 1 versus tertile 3: statistically significant, *p* < 0.05

^↑^Post hoc *p*: tertile 2 vs. tertile 3: statistically significant *p* < 0.05

^a^Proteinuria was defined as dipstick urinalysis result ≥ 1 +

### Outcomes of the index hospitalization and during follow-up

Of the 4,127 patients, 2,523 were admitted and six died in the ED. Patients with higher d-dimer levels were more likely to be admitted than those with lower d-dimer levels (45.1% vs. 60.3% vs. 78.0%, respectively, *p* < 0.001). However, the rates of ED revisits and readmissions did not differ significantly between groups within the first month of follow-up. However, the rates were higher in patients with higher d-dimer levels in the third month and at 1 year. Meanwhile, mortality rates at 1 month, 3 months, 1 year, and 3 years increased with increasing d-dimer levels (Table 2).

**Table 2:** Outcomes of the index visit to the emergency department and during the follow-up period according to d-dimer tertile

	All patients (*N* = 4,127)	Tertile 1^a^ (*n* = 1,376)	Tertile 2^b^ (*n* = 1,376)	Tertile 3^c^ (*n* = 1,375)	*p* value
Outcomes of the index visit to the ED					
Admission	2,523 (61.1)	620 (45.1)	830 (60.3)	1,073 (78.0)	< 0.001
Discharge	1,130 (27.4)	560 (40.7)	373 (27.1)	197 (14.3)	< 0.001
Discharge against medical advice	468 (11.3)	196 (14.2)	172 (12.5)	100 (7.3)	< 0.001
Death in the emergency department	6 (0.1)	0 (0.0)	1 (0.1)	5 (0.4)	0.03
Revisit to ED					
1-month revisit	309 (9.1)	94 (8.4)	106 (9.2)	109 (9.6)	0.583
3-months revisit	557 (16.4)	149 (13.3)	188 (16.4)	220 (19.5)	< 0.001
1-year revisit	984 (28.9)	262 (23.4)	341 (29.7)	381 (33.7)	< 0.001
Readmission					
1-month readmission	197 (5.8)	71 (6.3)	74 (6.4)	52 (4.6)	0.104
3-months readmission	299 (8.8)	95 (8.5)	105 (9.1)	99 (8.7)	0.856
1-year readmission	476 (14.0)	137 (12.2)	165 (14.3)	174 (15.3)	0.094
Mortality					
1-month mortality	213 (5.2)	5 (0.4)	35 (2.5)	173 (12.6)	< 0.001
3-months mortality	325 (7.9)	7 (0.5)	57 (4.1)	261 (19.0)	< 0.001
1-year mortality	590 (14.3)	20 (1.5)	129 (9.4)	441 (32.1)	< 0.001
3-year mortality	871 (21.1)	43 (3.1)	234 (17.0)	594 (43.2)	< 0.001

Data are presented as *n* (%)

*ED* Emergency department

^a^The range of d-dimer levels in tertile 1 was < 116 ng/mL

^b^The range of d-dimer levels in tertile 2 is 116–356 ng/mL

^c^The range of d-dimer level in tertile 3 was > 356 ng/mL

**Fig. 2:**
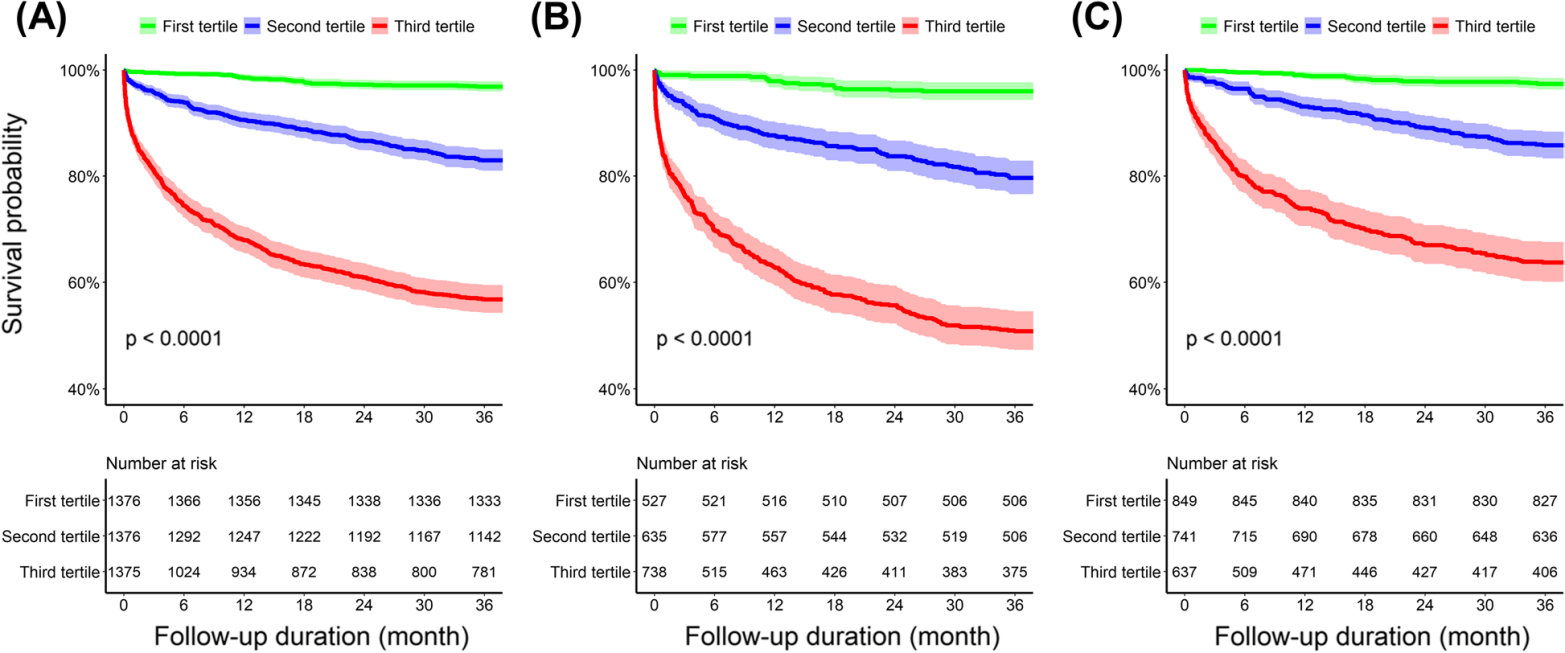
Kaplan–Meier curves for survival probability according to d-dimer tertiles in (**A**) overall patients, **B** patients with acute HMOD, and (**C**) patients without HMOD. HMOD, hypertension-mediated organ damage

Based on d-dimer level, survival probability was analyzed using the Kaplan–Meier method (Fig. 2). Among the three groups, the survival probability was significantly lower in patients with higher d-dimer levels (Fig. 2A). This trend was consistently observed in the subgroup analysis regardless of the presence or absence of acute HMOD (Fig. 2B, C). Table 3 shows the independent association between d-dimer level and all-cause mortality as determined by Cox proportional hazards regression analysis. Compared with patients in the lowest tertile of d-dimer level, those in the higher tertiles had a higher risk of 3-year all-cause mortality. After adjusting for factors such as sex, age, BP, comorbidities, and components of subclinical HMOD, patients in the second d-dimer tertile (adjusted HR, 2.847; 95% CI, 2.037–3.978), while those in the third (highest) d-dimer tertile (adjusted HR, 6.440; 95% CI, 4.628–8.961) had a significantly higher risk of 3-year all-cause mortality than those in the first d-dimer tertile.

**Table 3:** Hazard ratios for mortality according to d-dimer tertile

	Unadjusted HR (95% CI)	Model 1^a^ (95% CI)	Model 2^b^ (95% CI)	Model 3^c^ (95% CI)
All patients				
Tertile 1^*^	REF	REF	REF	REF
Tertile 2^†^	5.725 (4.135–7.925)	3.223 (2.310–4.498)	3.094 (2.216–4.321)	2.847 (2.037–3.978)
Tertile 3^‡^	18.290 (13.419–24.930)	8.506 (6.152–11.762)	7.595 (5.472–10.542)	6.440 (4.628–8.961)
Patients with acute HMOD				
Tertile 1^*^	REF	REF	REF	REF
Tertile 2^†^	5.486 (3.459–8.700)	3.177 (1.984–5.090)	3.036 (1.893–4.869)	2.939 (1.831–4.717)
Tertile 3^‡^	17.112 (11.018–26.575)	8.130 (5.144–12.849)	7.506 (4.727–11.918)	6.802 (4.266–10.846)
Patients without acute HMOD				
Tertile 1^*^	REF	REF	REF	REF
Tertile 2^†^	5.641 (3.563–8.932)	3.180 (1.981–5.105)	3.037 (1.892–4.875)	2.694 (1.675–4.334)
Tertile 3^‡^	17.629 (11.382–27.305)	8.381 (5.282–13.297)	7.358 (4.618–11.724)	6.215 (3.881–9.952)

*CI* Confidence interval, *HR* Hazard ratio, *REF* Reference

^*^The range of d-dimer levels in tertile 1 was < 116 ng/mL

^†^The range of d-dimer levels in tertile 2 is 116–356 ng/mL

^‡^The range of d-dimer level in tertile 3 was > 356 ng/mL

^a^Model 1 was adjusted for age, sex, and systolic and diastolic blood pressure

^b^Model 2 was adjusted for age, sex, systolic blood pressure, diastolic blood pressure, and comorbidities (hypertension, diabetes mellitus, dyslipidemia, ischemic stroke, hemorrhagic stroke, coronary artery disease, heart failure, and chronic kidney disease)

^c^Model 3 was adjusted for age, sex, systolic blood pressure, diastolic blood pressure, comorbidities (hypertension, diabetes mellitus, dyslipidemia, ischemic stroke, hemorrhagic stroke, coronary artery disease, heart failure, and chronic kidney disease), and components of hypertension-mediated organ damage (estimated glomerular filtration rate, hemoglobin level, cardiomegaly on chest radiography, left ventricular hypertrophy on electrocardiography, myocardial ischemia on electrocardiography, and atrial fibrillation on electrocardiography)

**Fig. 3:**
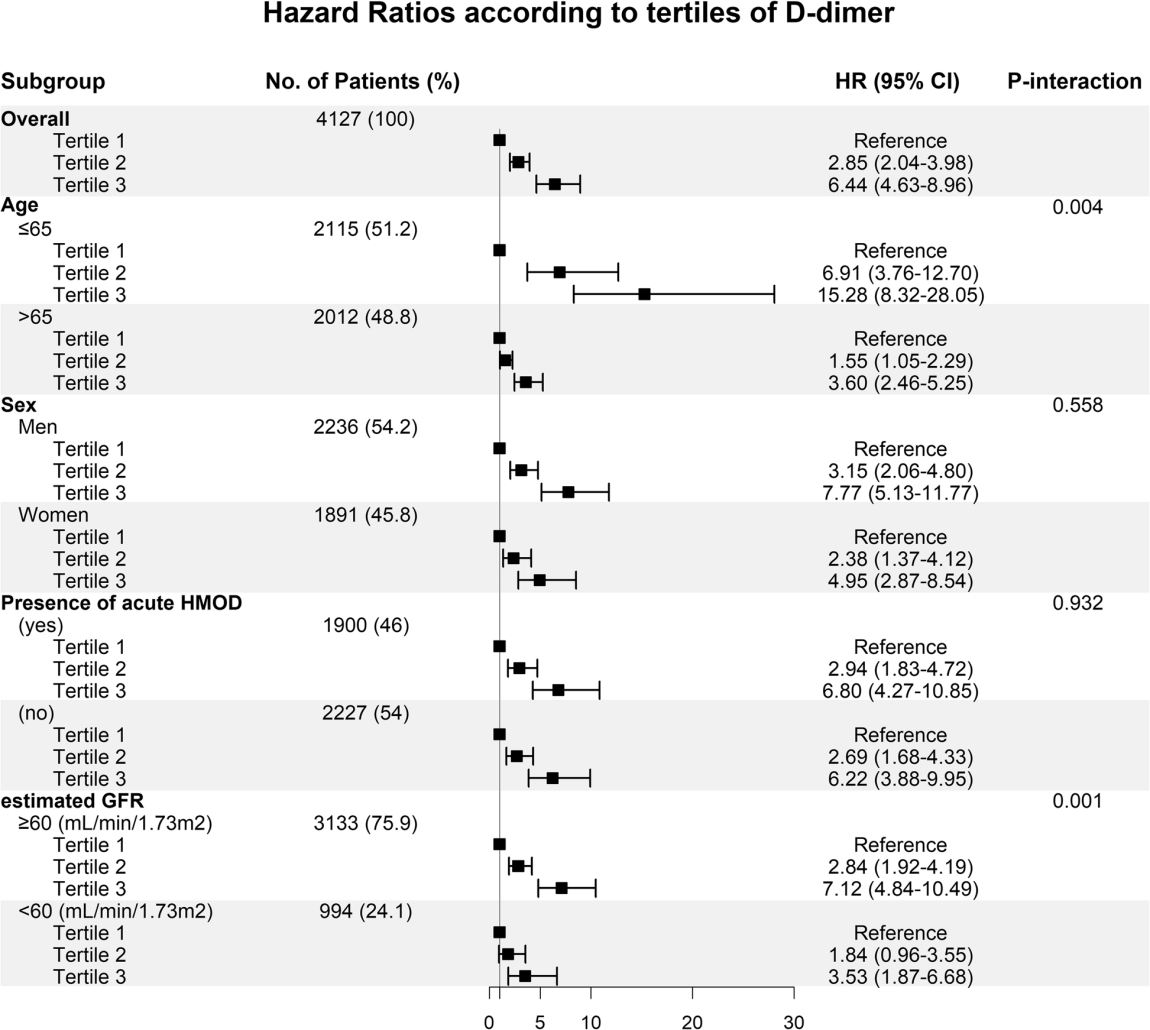
Subgroup analysis of risk of 3-year all-cause mortality according to d-dimer tertile using multivariable Cox proportional hazards regression models. Hazard ratios were adjusted for age, sex, systolic blood pressure, diastolic blood pressure, comorbidities (hypertension, diabetes mellitus, dyslipidemia, ischemic stroke, hemorrhagic stroke, coronary artery disease, heart failure, and chronic kidney disease), and components of hypertension-mediated organ damage (estimated glomerular filtration rate, hemoglobin, cardiomegaly on chest radiography, left ventricular hypertrophy on electrocardiography, and myocardial ischemia on electrocardiography, atrial fibrillation on electrocardiography). CI, confidence interval; eGFR, estimated glomerular filtration rate; HMOD, hypertension-mediated organ damage; HR, hazard ratio

In the subgroups with or without acute HMOD, patients in the second and third d-dimer tertiles also showed a significantly higher risk of 3-year all-cause mortality than those in the first tertile. The continuous association between d-dimer level and HR for 3-year all-cause mortality using restricted cubic spline curve analysis with full adjustment for confounders is presented in Fig. S1. The HR for 3-year all-cause mortality consistently increased with an increase in d-dimer level but showed a steep increase up to a d-dimer level of 300 ng/mL, followed by a gradual increase (*p* for non-linearity, < 0.001). We also performed a subgroup analysis that was stratified based on age (< 65 years or ≥ 65 years), sex (female or male), eGFR, and presence or absence of acute HMOD. The HR and 95% Cis for all-cause mortality according to d-dimer tertile were consistent across all subgroups (Fig. 3).

## Discussion

The main results of this study were as follows: (1) d-dimer levels were associated with a higher risk of all-cause mortality over a 3-year period independent of other clinically relevant variables in patients who presented with acute severe hypertension in the ED; (2) The risk of all-cause mortality increased in the group with higher d-dimer levels; and (3) An association between d-dimer level and all-cause mortality was consistently observed irrespective of acute HMOD.d-dimer represents a specific breakdown product of cross-linked fibrin clot formation and has been studied as a potential diagnostic marker for thromboembolic diseases such as deep vein thrombosis, pulmonary embolism, and disseminated intravascular coagulation. Circulating d-dimer levels are also elevated in patients with various other conditions including coronary artery disease, cancer, trauma, pregnancy, infectious and inflammatory diseases, severe renal disease, recent surgical procedures, advanced age, and many other diseases [27]. Increased d-dimer levels are reportedly associated with cardiovascular disease events and prognosis [23, 28].

Several studies have investigated the relationship between hypertension and d-dimer levels. Chi et al. demonstrated that hypertensive patients with LVH, left ventricular enlargement, and left atrial enlargement had higher d-dimer levels [29]. In addition, Sechi et al. reported that higher d-dimer levels were independently associated with advanced target organ damage in patients with hypertension [30]. A recent study revealed that d-dimer levels were higher in patients with hypertension than in controls; these levels increased significantly with hypertension severity [24]. However, few studies have assessed the association between d-dimer level and mortality in patients with acute severe hypertension.

Our study revealed a significant association between baseline d-dimer levels and mortality risk in patients with acute severe hypertension. Specifically, we found that patients in the second d-dimer level tertile (≥ 116.0 ng/mL) had a threefold increased risk of mortality. In addition, time-dependent receiver operating characteristic curve analysis showed that a d-dimer level > 250 ng/mL discriminated 3-year all-cause mortality with a sensitivity of 68.9% and specificity of 81.1% (area under the curve, 0.809; Fig. S2). Our findings suggest that even mildly elevated d-dimer levels may have a prognostic impact on mortality in patients with acute severe hypertension, indicating that increased d-dimer levels are associated with adverse outcomes. In addition, our study revealed a significant association between d-dimer levels and the risk of mortality, which persisted even in the subgroup of patients without acute HMOD. This finding suggests that an elevated d-dimer level may serve as a potential biomarker for subclinical HMOD in patients with acute severe hypertension. Thus, measuring d-dimer levels could have clinical implications for identifying high-risk groups that require a more comprehensive diagnostic approach, including in-depth examinations, timely and appropriate medical interventions, and follow-up strategies.

However, the mechanism by which d-dimer level predicts mortality remains unclear. Several mechanisms may explain these results. First, patients with higher d-dimer tertiles had more traditional cardiovascular risk factors and comorbidities, such as old age, hypertension, diabetes mellitus, chronic kidney disease, stroke, coronary artery disease, and heart failure as well as a higher frequency of proteinuria, cardiomegaly, and abnormal ECG findings than those in the lowest tertile. However, an association between d-dimer level and mortality risk was consistently observed, even after the adjustment for confounding factors. Second, given the mechanism of d-dimer formation, a higher d-dimer level may reflect greater systemic fibrin formation and a tendency toward increased thrombosis [16, 27]. A previous study reported that patients with essential hypertension have disequilibrium in the fibrinolytic system with a tendency toward a hypercoagulable state compared with normotensive subjects. This could partly explain the higher frequency of thrombotic complications in hypertensive patients than in normotensive subjects [31]. Therefore, patients with higher d-dimer levels are more likely to have more hypercoagulability associated with endothelial injury due to uncontrolled hypertension in patients with acute severe hypertension. Third, a previous study suggested that increased d-dimer levels represent increased coagulation and fibrinolytic activity and may provide clinical utility in predicting the risk of venous thromboembolism as well as future myocardial infarction, stroke, acute aortic dissection, or abdominal aortic aneurysm. Therefore, patients with higher d-dimer levels are more likely to have undiagnosed vascular diseases. Further studies are needed to clarify the mechanism underlying the association between d-dimer level and mortality risk in these populations.

This study has several potential limitations that should be considered when interpreting the results. First, this retrospective observational study was conducted at a single center, which may restrict the generalizability of our findings to other populations or settings. Second, we measured d-dimer levels only at the time of ED admission and did not perform follow-up with additional testing after discharge. Therefore, the predictive value of monitoring the changes in d-dimer levels over time with respect to mortality remains unclear. Third, our findings may have been influenced by unmeasured confounding variables. This study did not include information on anticoagulant therapy, which could be a relevant confounder. Despite our attempts to control multiple variables, other unknown confounders may have affected our results. Fourth, not all patients underwent diagnostic testing, including d-dimer assay, and it is possible that the testing frequency was higher in high- than low-risk patients, introducing selection bias (Table S2). Finally, the National Health Insurance Service did not provide the cause of death; therefore, we could not identify the cause of mortality or other adverse clinical events such as pulmonary embolism, sepsis, myocardial infarction, stroke, and cancer. A larger sample size, more d-dimer subgroups, more complete data, and longer follow-up periods are needed to further verify our findings.

Despite these limitations, this study has several strengths. To the best of our knowledge, it is the first extensive study to demonstrate a relationship between d-dimer level and long-term mortality in patients with acute severe hypertension. Our large dataset allowed us to account for potential confounders and revealed a clear dose–response relationship. Our study population is representative of a real-world cohort study. Our findings remained significant even after the adjustment for the most important biomarkers, including eGFR, hemoglobin, cardiomegaly and ECG findings. Additionally, complete coverage of the Korean population by the National Health Insurance Service ensures accurate and comprehensive records of patient mortality.

## Conclusions

The present study provides evidence that elevated d-dimer levels upon ED admission are significantly associated with an increased risk of all-cause mortality in patients with acute severe hypertension. d-dimer levels also have prognostic value in assessing the risk of mortality in these patients, which was consistently observed regardless of the presence of acute HMOD. These findings suggest that patients with elevated d-dimer levels require special attention and a more comprehensive therapeutic approach through an in-depth examination and close follow-up. Clinicians should pay more attention to patients with elevated d-dimer levels as they may have a high mortality risk.

## Supplementary Information

**Additional file 1: Supplementary Table S1.** Missing rate and comparison between the original data and imputed data. **Supplementary Table S2.** Comparison of included and excluded participants.

**Additional file 2: Fig. S1. **Continuous adjusted association between d-dimer and 3-year all-cause mortality using restricted cubic spline curve analysis. Hazard ratios were adjusted for age, sex, systolic blood pressure, diastolic blood pressure, comorbidities, and components of hypertension-mediated organ damage. The restricted cubic spline curve analysiswas performed using a d-dimer level of 250 ng/mL as the reference point.

**Additional file 3: Fig. S2. **Time-dependent receiver operating characteristic curve analysis for predicting all-cause mortality. Time-dependent receiver operating characteristic curve analysis was estimated using sensitivity and 1-specificity obtained from various cutoff levels of d-dimer at 3 years. AUC, area under the curve.

## Data Availability

The datasets used and analyzed in the current study are available from the corresponding author upon reasonable request.

## Abbreviations

BP: Blood pressure
CI: Confidence interval
DBP: Diastolic blood pressure
ECG: Electrocardiography
ED: Emergency department
eGFR: Estimated glomerular filtration rate
HMOD: Hypertension-mediated organ damage
HR: Hazard ratio
LVH: Left ventricular hypertrophy
SBP: Systolic blood pressure
VIF: Variable inflation factor
