# Supplementary material for: Association between D-dimer and long-term mortality in patients with acute severe hypertension visiting the emergency department
Source: Clin Hypertens. 2023 Jun 15;29:16. doi: 10.1186/s40885-023-00244-7 (PMC10268450; doi:10.1186/s40885-023-00244-7)
Supplement: Supplementary file 1 — Additional file 1: Supplementary Table S1. Missing rate and comparison between the original data and imputed data. Supplementary Table S2. Comparison of included and excluded participants. [file 40885_2023_244_MOESM1_ESM.docx]

| **Supplementary Table S1.** Missing rate and comparison between the original data and imputed data | | | | | |  |
| --- | --- | --- | --- | --- | --- | --- |
| Potential covariates for Cox proportional hazards regression analyses | Missing value, n (%) | Original data  (n = 4,127) | Imputed data  (n = 4,127) | *p*-value^*^ | SMD | |
| Age | 0 | 65 (53, 78) | 65 (53, 78) | 1 | <0.001 | |
| Women | 0 | 1,891 (45.8) | 1,891 (45.8) | 1 | <0.001 | |
| SBP, mmHg, median | 0 | 184 (165, 200) | 184 (165, 200) | 1 | <0.001 | |
| DBP, mmHg, median | 0 | 105 (100, 113) | 105 (100, 113) | 1 | <0.001 | |
| Hypertension | 95 (2.3) | 2,304 (57.1) | 2,350 (56.9) | 0.872 | 0.004 | |
| Diabetes mellitus | 139 (3.4) | 1,143 (28.7) | 1,181 (28.6) | 0.984 | 0.001 | |
| Dyslipidemia | 158 (3.8) | 453 (11.4) | 459 (11.1) | 0.704 | 0.009 | |
| Ischemic stroke | 157 (3.7) | 379 (9.5) | 385 (9.3) | 0.766 | 0.007 | |
| Hemorrhagic stroke | 164 (4.0) | 129 (3.3) | 132 (3.2) | 0.935 | 0.003 | |
| Coronary artery disease | 158 (3.8) | 464 (11.7) | 473 (11.5) | 0.773 | 0.007 | |
| Heart failure | 164 (4.0) | 234 (5.9) | 235 (5.7) | 0.721 | 0.009 | |
| Chronic kidney disease | 158 (3.8) | 368 (9.3) | 376 (9.1) | 0.832 | 0.006 | |
| eGFR, mL/min/1.73 m2 | 4 (0.1%) | 85 (61, 99) | 85 (61, 99) | 0.996 | <0.001 | |
| Hb, g/dL | 4 (0.1%) | 13.7 (12.2, 15.0) | 13.7 (12.2, 15.0) | 0.980 | <0.001 | |
| Cardiomegaly on chest radiography | 165 (4.0%) | 576 (14.5) | 590 (14.3) | 0.781 | 0.007 | |
| LVH on ECG | 194 (4.7%) | 448 (11.4) | 460 (11.1) | 0.755 | 0.008 | |
| Myocardial ischemia on ECG | 194 (4.7%) | 355 (9.0) | 363 (8.8) | 0.746 | 0.008 | |
| Atrial fibrillation on ECG | 194 (4.7%) | 281 (7.1) | 282 (6.8) | 0.614 | 0.012 | |

Data are presented as n (%) or median (IQR) as appropriate.

SMD, standardized mean differences; IQR, interquartile range; SBP, systolic blood pressure; DBP, diastolic blood pressure; eGFR, estimated glomerular filtration rate; Hb, hemoglobin; LVH, left ventricular hypertrophy; ECG, electrocardiography.

^*^Categorical variables were compared using the chi-squared test or Fisher's exact test, whereas continuous variables were compared using the Mann–Whitney *U* test.

| **Supplementary Table S2.** Comparison of included and excluded participants | | | |  |
| --- | --- | --- | --- | --- |
|  | Included  (n = 4,127) | Excluded  (n = 6,092) | *p*-value | |
| Age | 65 (53, 78) | 53 (42, 65) | <0.001 | |
| Women | 1,891 (45.8) | 2,938 (48.2) | 0.018 | |
| Medical history |  |  |  | |
| Hypertension | 2,304 (57.1) | 2,202 (38.1) | <0.001 | |
| Diabetes mellitus | 1,143 (28.7) | 985 (17.2) | <0.001 | |
| Dyslipidemia | 453 (11.4) | 467 (8.3) | <0.001 | |
| Ischemic stroke | 379 (9.5) | 258 (4.6) | <0.001 | |
| Hemorrhagic stroke | 129 (3.3) | 116 (2.1) | <0.001 | |
| Coronary artery disease | 464 (11.7) | 267 (4.7) | <0.001 | |
| Heart failure | 234 (5.9) | 81 (1.4) | <0.001 | |
| Chronic kidney disease | 368 (9.3) | 196 (3.5) | <0.001 | |
| End-stage renal disease | 182 (4.6) | 93 (1.7) | <0.001 | |
| Social history |  |  |  | |
| Cigarette smoking | 814 (27.1) | 782 (25.1) | <0.001 | |
| Alcohol consumption | 1,078 (35.7) | 1,263 (38.9) | 0.008 | |
| Triage vitals |  |  |  | |
| SBP, mmHg | 184 (165, 200) | 172 (158, 187) | <0.001 | |
| DBP, mmHg | 105 (100, 113) | 104 (100, 111) | 0.314 | |
| Laboratory tests |  |  |  | |
| eGFR, mL/min/1.73 m2 | 85 (61, 100) | 97 (80, 109) | <0.001 | |
| Hb, g/dL | 13.7 (12.2, 15.0) | 14.0 (12.9, 15.3) | <0.001 | |
| BNP, pg/mL | 75 (26, 282) | 67 (25, 177) | 0.061 | |
| Troponin-I, ng/mL | 0.01 (0.01, 0.03) | 0.01 (0.01, 0.01) | <0.001 | |
| Urinary analysis |  |  |  | |
| Proteinuria^a^ | 931 (34.0) | 691 (25.2) | <0.001 | |
| Chest radiography |  |  |  | |
| Cardiomegaly | 576 (14.5) | 411 (9.6) | <0.001 | |
| Electrocardiography |  |  |  | |
| LVH | 448 (11.4) | 301 (9.1) | 0.002 | |
| Myocardial ischemia | 355 (9.0) | 127 (3.8) | <0.001 | |
| Atrial fibrillation | 281 (7.1) | 95 (2.9) | <0.001 | |
| Acute HMOD | 1,900 (46.0) | 606 (9.9) | <0.001 | |
| Outcomes of the index visit to the ED |  |  |  | |
| Admission | 2,523 (61.1) | 1,613 (26.5) | <0.001 | |
| Discharge | 1,130 (27.4) | 3,985 (65.4) | <0.001 | |
| Discharge against medical advice | 468 (11.3) | 491 (8.1) | <0.001 | |
| Death in the emergency department | 6 (0.1) | 3 (0.0) | 0.205 | |
| Revisit to ED |  |  |  | |
| 1-month revisit | 309 (9.1) | 416 (9.8) | 0.323 | |
| 3-months revisit | 557 (16.4) | 681 (16.0) | 0.684 | |
| 1-year revisit | 984 (28.9) | 1,185 (27.9) | 0.310 | |
| Readmission |  |  |  | |
| 1-month readmission | 197 (5.8) | 259 (6.1) | 0.618 | |
| 3-months readmission | 299 (8.8) | 374 (8.8) | 1 | |
| 1-year readmission | 476 (14.0) | 543 (12.7) | 0.124 | |
| Mortality |  |  |  | |
| 1-month mortality | 213 (5.2) | 95 (1.6) | <0.001 | |
| 3-months mortality | 325 (7.9) | 164 (2.7) | <0.001 | |
| 1-year mortality | 590 (14.3) | 311 (5.1) | <0.001 | |
| 3-year mortality | 871 (21.1) | 547 (9.0) | <0.001 | |

Data are presented as *n* (%) or median (IQR), as appropriate.

IQR, interqurtile range; SBP, systolic blood pressure; DBP, diastolic blood pressure; eGFR, estimated glomerular filtration rate; Hb, hemoglobin; BNP, B-type natriuretic peptide; LVH, left ventricular hypertrophy; HMOD, hypertension-mediated organ damage.

^a^Proteinuria was defined as dipstick urinalysis result ≥ 1+.
