# Supplementary figures and images for: Association between D-dimer and long-term mortality in patients with acute severe hypertension visiting the emergency department
Source: Clin Hypertens. 2023 Jun 15;29:16. doi: 10.1186/s40885-023-00244-7 (PMC10268450; doi:10.1186/s40885-023-00244-7)

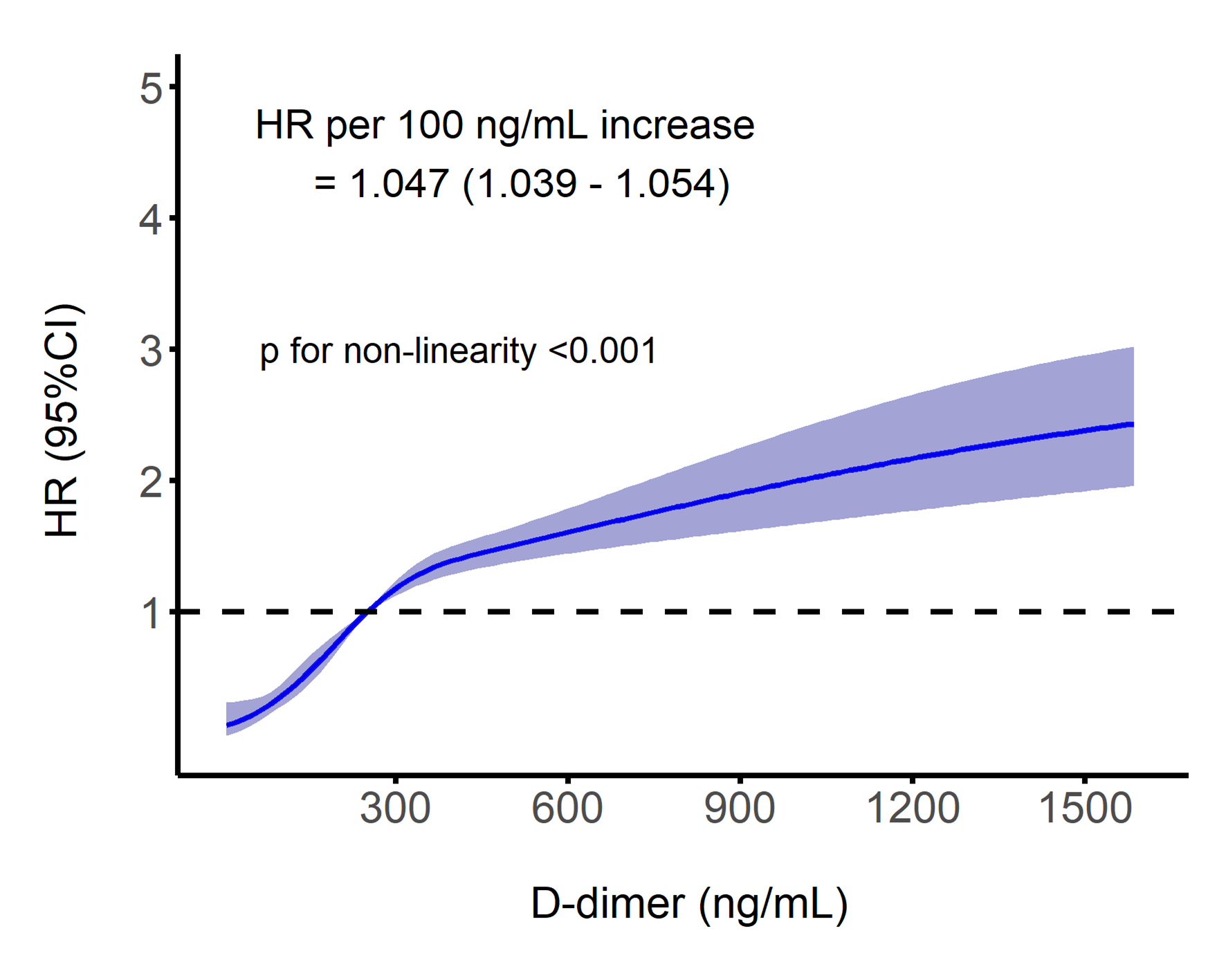

Supplement: Supplementary file 2 — Additional file 2: Fig. S1. Continuous adjusted association between d-dimer and 3-year all-cause mortality using restricted cubic spline curve analysis. Hazard ratios were adjusted for age, sex, systolic blood pressure, diastolic blood pressure, comorbidities, and components of hypertension-mediated organ damage. The restricted cubic spline curve analysiswas performed using a d-dimer level of 250 ng/mL as the reference point. [file 40885_2023_244_MOESM2_ESM.tif]

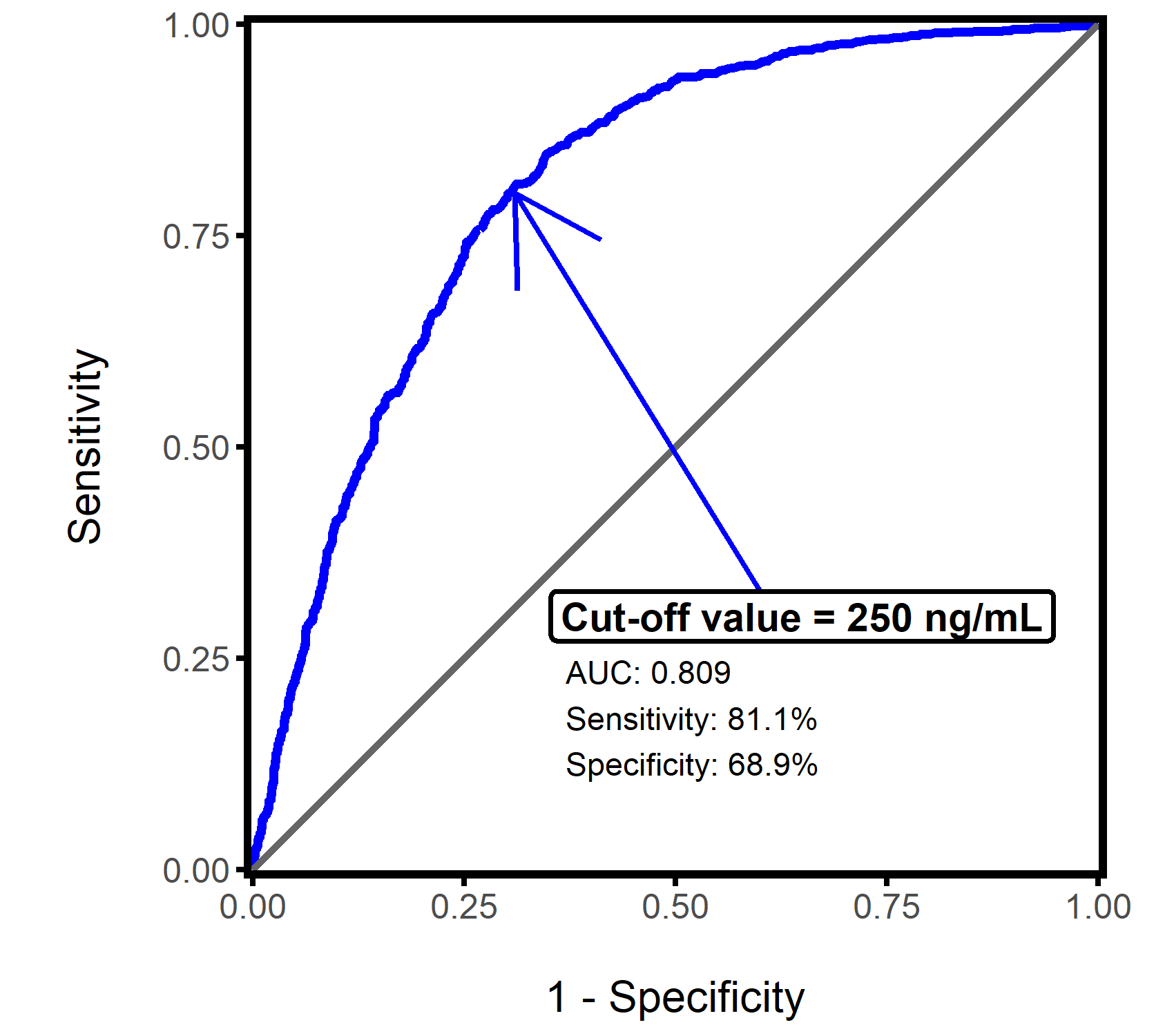

Supplement: Supplementary file 3 — Additional file 3: Fig. S2. Time-dependent receiver operating characteristic curve analysis for predicting all-cause mortality. Time-dependent receiver operating characteristic curve analysis was estimated using sensitivity and 1-specificity obtained from various cutoff levels of d-dimer at 3 years. AUC, area under the curve. [file 40885_2023_244_MOESM3_ESM.tif]
